# Assessment of Atmospheric Algorithms to Retrieve Vegetation in Natural Protected Areas Using Multispectral High Resolution Imagery

**DOI:** 10.3390/s16101624

**Published:** 2016-09-30

**Authors:** Javier Marcello, Francisco Eugenio, Ulises Perdomo, Anabella Medina

**Affiliations:** Instituto de Oceanografía y Cambio Global (IOCAG), Universidad de Las Palmas de Gran Canaria (ULPGC), Campus Universitario de Tafira, 35017 Las Palmas, Gran Canaria, Spain; francisco.eugenio@ulpgc.es (F.E.); ulises.perdomo101@alu.ulpgc.es (U.P.); anabella.medina101@alu.ulpgc.es (A.M.)

**Keywords:** atmospheric correction, ATCOR, FLAASH, 6S, semi-arid ecosystems, high resolution WorldView-2 images

## Abstract

The precise mapping of vegetation covers in semi-arid areas is a complex task as this type of environment consists of sparse vegetation mainly composed of small shrubs. The launch of high resolution satellites, with additional spectral bands and the ability to alter the viewing angle, offers a useful technology to focus on this objective. In this context, atmospheric correction is a fundamental step in the pre-processing of such remote sensing imagery and, consequently, different algorithms have been developed for this purpose over the years. They are commonly categorized as imaged-based methods as well as in more advanced physical models based on the radiative transfer theory. Despite the relevance of this topic, a few comparative studies covering several methods have been carried out using high resolution data or which are specifically applied to vegetation covers. In this work, the performance of five representative atmospheric correction algorithms (DOS, QUAC, FLAASH, ATCOR and 6S) has been assessed, using high resolution Worldview-2 imagery and field spectroradiometer data collected simultaneously, with the goal of identifying the most appropriate techniques. The study also included a detailed analysis of the parameterization influence on the final results of the correction, the aerosol model and its optical thickness being important parameters to be properly adjusted. The effects of corrections were studied in vegetation and soil sites belonging to different protected semi-arid ecosystems (high mountain and coastal areas). In summary, the superior performance of model-based algorithms, 6S in particular, has been demonstrated, achieving reflectance estimations very close to the in-situ measurements (RMSE of between 2% and 3%). Finally, an example of the importance of the atmospheric correction in the vegetation estimation in these natural areas is presented, allowing the robust mapping of species and the analysis of multitemporal variations related to the human activity and climate change.

## 1. Introduction

Mapping vegetation in semi-arid areas is a challenging undertaking. These environments usually contain sparse vegetation with small species having a reduced leaf area that limits the applicability of certain vegetation indices. Furthermore, the important soil reflectance contribution makes it difficult to obtain precise information about the types of vegetation [1].

The launch of new satellites, with improved capabilities, can assist in the generation of precise vegetation maps in natural protected areas. In this context, Worldview-2 and 3 offer a very high spatial resolution with additional bands not included in previous high resolution platforms (i.e., Ikonos, Quickbird, Geoeye, Quickbird, KOMPSAT, Pleiades, etc.) in the visible and near infrared spectrum. However, the spectral data acquired by satellite sensors are influenced by atmospheric absorption and scattering, which results in a distortion of the actual reflectance of the different covers and, consequently, affects the precise extraction of information from the imagery. As previously mentioned, the emergence of high-resolution satellites with new spectral channels and the ability to change their viewing angle has highlighted the importance of properly modeling the atmospheric effects.

As indicated, the radiance recorded at the sensor is not fully representative of the Earth’s surface as it is altered by the atmosphere through which electromagnetic energy has to pass. Therefore, atmospheric correction is an important pre-processing step in many applications. It is critical in the monitoring of littoral zones (i.e., water quality, bathymetry or seafloor mapping) due to the low radiation reaching the sensor from water areas as a consequence of the low reflectivity of the water. In these scenarios, the major contribution to the signal comes from the atmosphere. In land areas, it is also important to obtain the real reflectance from the soil and vegetation if biophysical parameters are going to be extracted (i.e., biomass, leaf area index, etc.) or to properly achieve precise Land Use Land Cover or vegetation maps. A major benefit is when dealing with multitemporal remotely sensed imagery to overcome change detection analysis or phenological studies [2].

To compensate for atmospheric effects, parameters such as the distribution of aerosols, amount of water vapor and scene visibility must be known. Different strategies have been developed as direct measurements of these atmospheric properties are rarely available. Commonly used image-based atmospheric correction methods are Dark Object Subtraction (DOS) [3], that corrects for the additive scattering effect; Cosine of the sun zenith angle COST [4], that also takes into account the multiplicative transmittance effect; and QUick Atmospheric Correction (QUAC) [5], that is based on the empirical finding that the average reflectance of diverse material spectra is not dependent on each scene. The Simplified Method for Atmospheric Correction (SMAC) [6] is a semi-empirical method designed for the atmospheric correction of the large series of data acquired by large field of view sensors. The Empirical Line Calibration (ELC) [7] uses field-measured surface reflectance from a series of invariant-in-time calibration targets to estimate the reflectance for each band. A regression equation is then developed for each band of an image to carry out the atmospheric correction. Other more complex approaches model the atmosphere which usually use an accurate radiative transfer code (RTC) to correct the atmospheric effects, such as the MODerate resolution atmospheric TRANsmission (MODTRAN) [8,9], the Fast Line-of-sight Atmospheric Analysis of Spectral Hypercubes (FLAASH) [10], the ATmospheric CORrection (ATCOR) [11,12] and the Second Simulation of a Satellite Signal in the Solar Spectrum (6S) [13,14] models.

There has been some research comparing different approaches to correct the atmospheric effects [15,16,17,18,19,20,21,22,23,24,25,26,27]. In this context, Wu et al. (2005) [16] only addressed image-based algorithms using QuickBird imagery. Mahiny and Turner (2007) [17] carried out a study of four atmospheric correction methods on Landsat scenes, but two of the methods were relative approaches (PIF and RCS). El Hajj et al. (2008) [18] presented an atmospheric correction method based on the 6S model and applied it to SPOT 5 data. Martin et al. (2012) [22] considered three methods in the analysis (DOS, COST and 6S). Pacifici (2013) [23] and Agrawal and Sarup (2011) [20] compared FLAASH and QUAC. Vanonckelen et al. (2013) [24] analyzed the effect of atmospheric and topographic correction strategies on the accuracy of land cover classification, however only two atmospheric techniques were compared. Broszeit and Ashraf (2013) [25] compared COTS and ATCOR using Geoeye and Rapideye data. More recently, Nguyen et al. (2015) [26] analyzed three models (DOS, FLAASH and 6S) using medium resolution data. Pu et al. (2015) [27] evaluated two methods (ELC and FLAASH) and a combination of both to identify urban tree species with WorldView-2 (WV-2) imagery.

After this review of the literature, we found that the studies were not extensive in the sense that they mainly covered two or three methods, were usually not applied to 8-band high resolution satellite data, did not analyze semi-arid regions, or did not assess the influence of the configuration parameters in detail. This work presents a detailed study of five atmospheric correction algorithms (DOS, QUAC, FLAASH, ATCOR and 6S) applied to the eight bands of the high resolution Worldview-2 satellite. After reviewing the state-of-the-art in atmospheric correction methodologies, five algorithms were selected as they are widely used in remote sensing and they are representative of each strategy. The effects of the corrections were studied in two representative semi-arid natural protected areas (mountain and coastal ecosystems). This study also includes a detailed analysis of the influence of parameterization on the estimated surface reflectance of the atmospheric correction based on RTC models. In-situ spectral data were collected simultaneously to the satellite overflight to evaluate the results. Finally, the atmospheric correction influence in the vegetation estimation and mapping is presented.

Section 2 includes the areas of interest, the available field data and the satellite imagery used in the analysis. Next, the atmospheric correction algorithms and the methodology applied are described. Section 3 presents and discusses the results of the study and provides some insights into the mapping of vegetation. Finally, the conclusions are summarized in the Section 4.

## 2. Materials and Methods

### 2.1. Semi-Arid Ecosystems Analyzed

**Figure 1:**
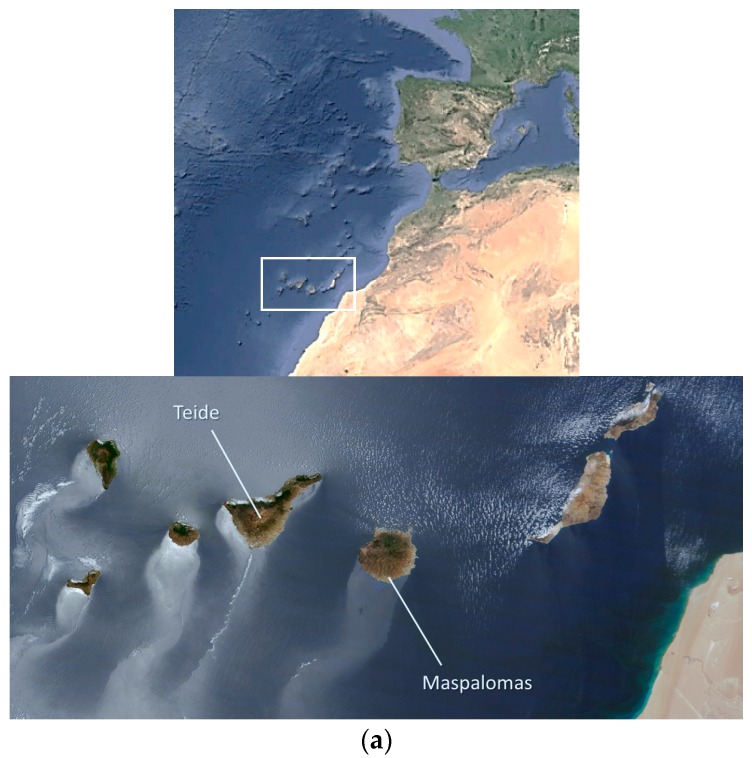

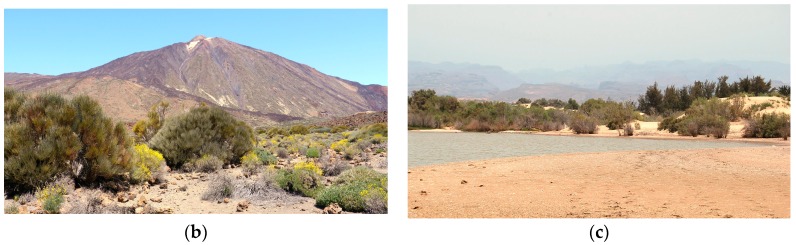
Natural protected areas: (**a**) Location of the Canary Islands; (**b**) Teide National Park; (**c**) Maspalomas Special Natural Reserve.

Two protected areas located in the Canary Islands (Spain) have been selected as representative semi-arid ecosystems (see Figure 1), considerably threatened by human presence due to the intense touristic activity: the Teide National Park (Tenerife Island) and the Maspalomas Special Natural Reserve (Gran Canaria Island).

The Teide National Park was created in 1954 to protect this remarkable landscape of great ecological importance which lies at the base of the colossal volcano (3718 m high), covering an area of 18,990 ha. It includes plant species that are unique and adapted to the rough environmental conditions such as high altitude, intense sunlight and extreme temperature variations. Specifically, a total of 222 plants grow there. Plants respond to thermic and hydric stress with a shrub physiognomy. The Teide broom (*Spartocytisus supranubius*) is one of the most important plant species, as are Rosalillo de cumbre (*Pterophalus lasiospermus*) and Hierba pajonera (*Descurania bourgaeana*) [28]. Another important species is the Canary pine (*Pinus canariensis*) that appears in the northern area of the Park, being the only native pine on the archipelago [29].

The Maspalomas area was protected in 1987 and it covers 403.9 ha of sand dunes, endemic vegetation and a lagoon of great ecological value. This reserve is very complex as it presents a great variety of natural systems (marine, eolic, lake and fluvial). Moreover, the tourism and urbanization development has conditioned the natural evolution of the system, interfering with the sand dynamics. Thus, the sedimentary deposition has had a deficit since the 1960’s. It is important to protect this ecosystem due to the presence of threatened habitats, its enormous ornithological interest, the threatened species of flora and fauna and to preserve the geomorphological structures. Vegetation can be described as xerocanaria from a deserted habitat with halophilic (salt excess) and psammophilic (sand sediment) species. However, some hydrophilic species can be found next to the lagoon. Some representative plants living in the dune system include *Tamarix canariensis*, *Juncus acutus*, *Launeaea Arborecens*, *Traganum moquinii* or *Cyperus laevigatus* [30].

### 2.2. Field Data

In-situ field data in the Maspalomas natural area were acquired simultaneously to the Worldview-2 imagery on 4 June 2015. In addition, a campaign in the Teide National Park was conducted on 5 June 2015. A total of 28 sites were monitored covering different types of soils and vegetation species. In order to describe the different land cover classes, the following information was collected at each site to provide ground reference data for the analysis: the ground reflectivity at different wavelengths was recorded using the ADS Fieldspec 3 spectroradiometer; the precise location coordinates and time were collected using a GPS receiver; the solar azimuth and zenithal angles were recorded and the ozone column, the total water vapor and the aerosol optical thickness was measured using the handheld MICROTOPS II sun-photometer.

**Figure 2:**
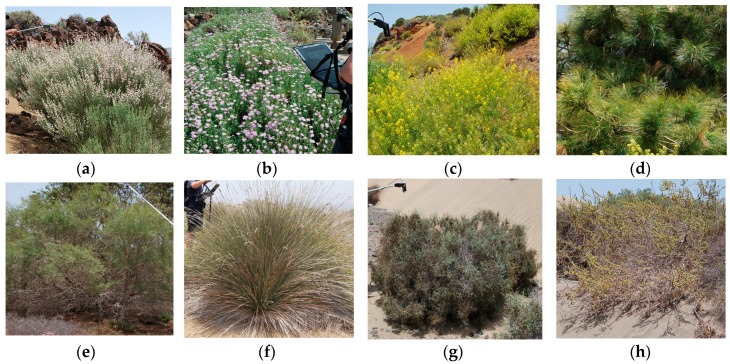
Vegetation measured at the Teide Park: (**a**) *Spartocytisus supranubius*; (**b**) *Pterophalus lasiospermus*; (**c**) *Descurania bourgaeana*; (**d**) *Pinus canariensis*. Vegetation measured at Maspalomas: (**e**) *Tamarix canariensis*; (**f**) *Juncus acutus*; (**g**) *Launaea arborecens*; (**h**) *Traganum moquinii*.

In order to obtain measurements comparable to those from the satellite, at each site, several radiance measurements were acquired at different angles, apart from the nadir, to better characterize the plant canopy and soil spectral response. In addition, to better account for the spectral variability of the vegetation, sampling was carried out by selecting plants, for each species, and, when possible, at different phenological stages. Examples of some species monitored during the field campaign at the Teide Park and Maspalomas Reserve are shown in Figure 2.

Reflectance measurements were recorded in the visible and near-infrared range of the spectrum (350–2500 nm) and the field spectroscopy metadata was organized based on ISO and OGC standards [31]. Reflectance measurements were carried out over homogeneous and flat areas.

### 2.3. Remote Sensing Imagery

**Figure 3:**
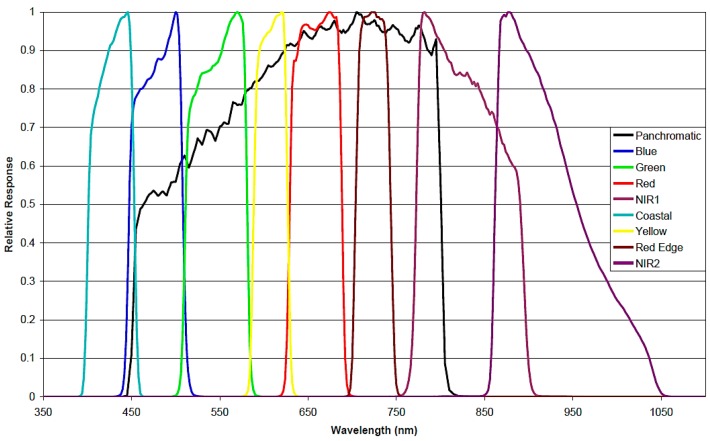
Worldview-2 relative spectral radiance response (nm) [32].

WorldView-2, manufactured by ITT Space Systems Division for DigitalGlobe (Westminster, CO, USA), was declared operational on 4 January 2010. Its sensor has a spatial resolution of 0.46 m and 1.8 m at the nadir for the panchromatic and multispectral (MS) bands, respectively. The eight MS bands are: coastal (400–450 nm), blue (450–510 nm), green (510–580 nm), yellow (585–625 nm), red (630–690 nm), red edge (705–745 nm), NIR1 (770–895 nm), and NIR2 (860–1040 nm). The spectral response of each band is shown in Figure 3. Its nominal swath width is 16.4 km and the radiometric resolution is 11 bits. WV-2 satellite provides finer spatial resolution and more spectral information in the visible spectrum than previous high resolution satellites. However, producing validated products requires a number of challenges in the pre-processing steps to be overcome.

**Figure 4:**
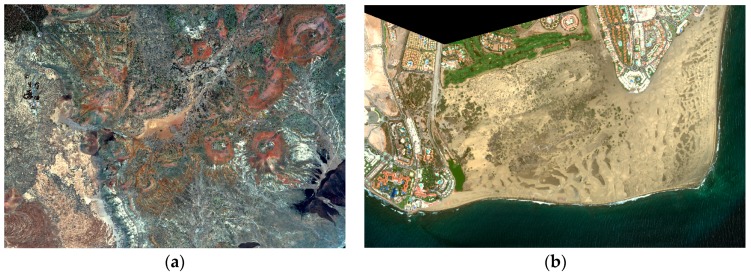
Worldview-2 satellite imagery used: (**a**) Teide National Park; (**b**) Maspalomas Special Natural Reserve.

Figure 4 includes the Worldview-2 color composite imagery used in the analysis. In particular, the Teide National Park image was acquired on 16 May 2011 and the Maspalomas Natural Reserve image was sensed on 4 June 2015.

### 2.4. Atmospheric Correction Models

There are a number of atmospheric correction methods and algorithms which can be categorized as image-based and physical model-based. The following 5 representative algorithms have been selected for the analysis: DOS, QUAC, FLAASH, ATCOR and 6S.

The DOS method [3] is a simple image-based technique to remove the additive scattering component caused by path radiance. The basic assumption is that few targets on the Earth’s surface are in complete shade and, consequently, their received radiances are due to atmospheric scattering. Therefore, the equation to obtain the surface reflectivity is based on:

(1) $$\rho_{SUP}=\frac{d^{2}\pi \left(L_{TOA}-L_{o}\right)}{\left(E_{TOA}\cos\theta_{i}\right)},$$

where *d* is the Earth-Sun distance, $L_{TOA}$ is the spectral radiance at satellite’s sensor, $L_{o}$ is the upwelling atmospheric spectral radiance scattered (based on the minimum values of the histogram for the separate spectral bands), $E_{TOA}$ is the solar spectral irradiance on a surface perpendicular to the Sun’s rays outside the atmosphere and $\theta_{i}$ is the solar zenith angle.

The QUAC algorithm [5] determines atmospheric compensation parameters directly from the information contained within the scene. The QUAC principle is essentially the determination of an offset and a gain parameter, in order to retrieve the surface reflectance. It is expressed by:

(2) $$\rho_{SUP}=Gain\left(L_{TOA}-Offset\right),$$

where *Offset* is the lowest reflectance value for each channel and defines the baseline spectrum and *Gain* is the ratio between the average of the endmember spectra representing a reference library of material reflectance spectra and the average of a collection of endmembers retrieved from the observed, in-scene pixel spectra.

The FLAASH [10] atmospheric correction code derives its physics-based algorithm from the MODTRAN-4 radiative transfer code. FLAASH is designed to eliminate atmospheric effects caused by molecular and particulate scattering and absorption from the radiance at the sensor and to obtain reflectance at the surface. It is determined by:

(3) $$L_{TOA}=\left(\frac{A\rho_{SUP}}{1-\rho_{e}S}\right)+\left(\frac{B\rho_{e}}{1-\rho_{e}S}\right)+L_{o},$$

where $\rho_{SUP}$ is the pixel surface reflectance, $\rho_{e}$ is an average surface reflectance for the pixel and the surrounding region, *S* is the spherical albedo of the atmosphere, $L_{o}$ is the radiance backscattered by the atmosphere and *A* and *B* are coefficients that depend on atmospheric and geometric conditions. The first term in Equation (3) corresponds to the radiance from the surface that travels directly into the sensor, while the second term corresponds to the radiance from the surface that is scattered by the atmosphere into the sensor. The distinction between $\rho_{SUP}$ and $\rho_{e}$ accounts for the “adjacency effect” (spatial mixing of radiance among nearby pixels) caused by atmospheric scattering. The values of A, B, S, and $L_{o}$ can be determined empirically from the standard MODTRAN-4 simulations for a specified atmosphere model (Rural, Urban, Maritime, Tropospheric). The viewing and solar angles of the measurement and nominal values for the surface elevation, aerosol type and visible range for the scene must be specified.

The ATCOR [12] algorithm is also based on the radiative transfer theory developed in MODTRAN-4. The correction procedure in mainly divided into two steps: the first involves getting the atmospheric effect assuming an isotropic or Lambertian reflectance law, without taking the adjacency effect into account. Meanwhile, the second step models the reflected radiation from the neighborhood considering the adjacency radiance.

The surface reflectance disregarding the adjacency component is obtained by Equation (4). To obtain the $a_{0}$ and $a_{1}$ coefficients, an accurate estimation of the main atmospheric parameters (aerosol type, visibility or optical thickness and water vapor column) is necessary:

(4) $$\rho_{SUP}=\frac{1}{a_{1}}\left(\frac{d^{2}\pi L_{TOA}}{E_{TOA}\cos\theta_{i}}-a_{0}\right).$$

The average reflectivity over the target area ${{\bar{\rho }}_{SUP}}_{i}$ is computed to correct the adjacency effect. Thus, Equation (5) describes the relationship to get the surface reflectance free of the adjacency effect:

(5) $$\rho_{SUP}^{\prime }=\rho_{SUP}+\left(\int_{\lambda_{1}}^{\lambda_{2}}\frac{\tau_{o_{dif}}}{\tau_{o_{dir}}}Rd\lambda \right)\left(\rho_{SUP}-\overset{n_{R}}{\underset{i=1}{\sum }}{{\bar{\rho }}_{SUP}}_{i}w_{i}\right),$$

where, $\tau_{o_{dif}}$ and $\tau_{o_{dir}}$ are the diffuse and direct transmittance respectively, *R* is the sensor-specific spectral response curve and $w_{i}$ defines weighting coefficients as a distance-dependent function.

6S [13] is a radiative transfer code designed to predict the reflectance at the top of the atmosphere simulating the atmospheric conditions. The model generates *x_a_*, *x_b_* and *x_c_* constants to estimate the surface reflectance free of atmospheric effect, through Equations (6) and (7):

(6) $$\rho_{SUP}=\frac{y}{1+\left(x_{c}y\right)},$$

(7) $$y=\left(x_{a}L_{TOA}\right)-x_{b}.$$

To remove the adjacency effect, the following improvement was implemented:

(8) $$\rho_{SUP}^{\prime }=\rho_{SUP}+\frac{\tau_{o_{dif}}}{\tau_{o_{dir}}}\left[\rho_{SUP}-\bar{\rho }\right],$$

where $\rho_{SUP}^{\prime }$ is the surface reflectivity considering the adjacency effect, $\rho_{SUP}$ is the corrected surface reflectivity by the initial 6S model, $\tau_{o_{dif}}$ and $\tau_{o_{dir}}$ are the diffuse and direct transmittances and $\bar{\rho }$ is the average reflectivity contribution from the pixel background.

### 2.5. Comparison Methodology

In this study, two strategies have been used to compare the atmospheric correction methods: relative and absolute evaluation.

The first one consists of a relative evaluation, changing the input factors (parameters and models) in the configuration to identify how they affect in the estimation of the surface reflectivity. Obviously, only model-based algorithms were considered. Specifically, 5 different inputs were adjusted in the analysis: atmospheric model, aerosol model, aerosol optical thickness, adjacency effect and altitude. The water vapor content was not assessed as it is accounted for in the atmospheric model and altitude.

**Figure 5:**
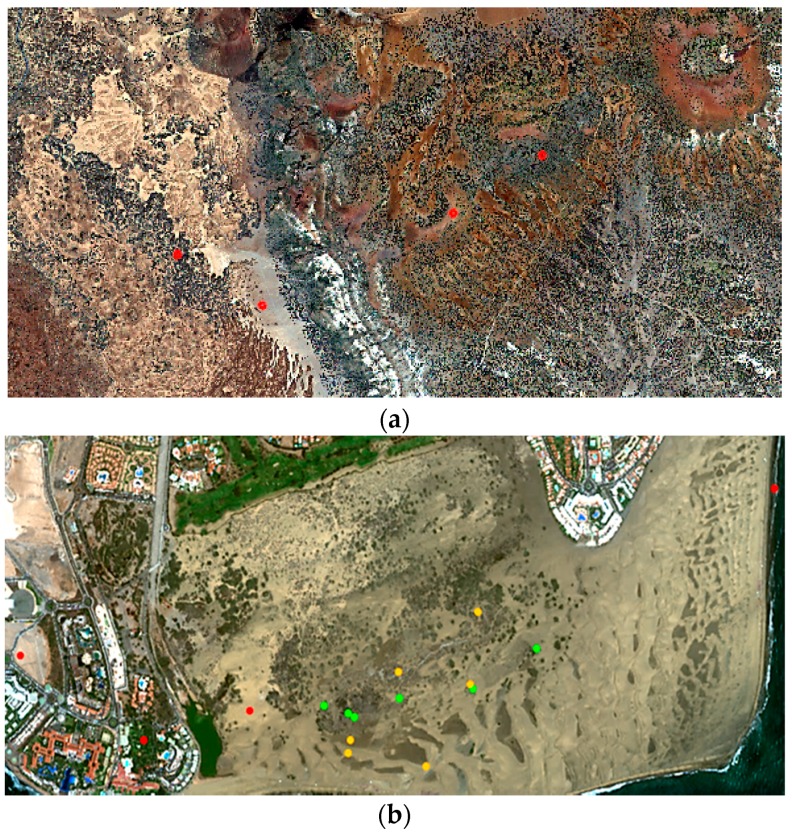
Sites analyzed (red for the relative analysis and green and orange for the absolute evaluation of vegetation and soil points): (**a**) Teide National Park; (**b**) Maspalomas Natural Reserve.

The second strategy compares the reflectivity of the atmospherically corrected image pixels with the field measurements recorded using the ADS Fieldspec 3 spectroradiometer. Representative vegetation and soil sites included in the analysis are shown in Figure 5. Special care was taken into account to generate the match-ups between field and satellite locations in order to guarantee that we are comparing the same sites. Therefore, each specific plant measured was identified in the WV-2 image around the GPS location while, for the bare soil, large, flat and homogeneous areas were sampled and, consequently, the comparison will be accurate even if errors of few meters between satellite and ground measurements could be present (4.5 m CE90 is the expected location accuracy for the WV-2 imagery used). The Root-Mean-Square Error (RMSE) was computed to quantify the estimated reflectivity differences and the BIAS was used to determine the tendency to overestimate or underestimate as regards field data.

After the relative assessment (Section 3.1) and considering the information from the area under analysis and the technical specifications of the correction models, the effect of each input in the estimated reflectance obtained by each atmospheric correction algorithm was analyzed and, consequently, the most appropriate factors were identified. Table 1 summarizes the corresponding input settings used in the comparative analysis with the field data. A total of 4 scenarios (A, B, C and D) have been considered. They fix the atmospheric model (the one selected is the most appropriate for the region) but change the aerosol model, the aerosol optical thickness (AOT) (based on the measured and satellite-derived values) and the consideration of the adjacency effect from neighboring pixels. The altitude of the scene is also fixed and set to the mean value as this information is well-known and, within the scenes considered, altitude changes are minimal.

### 2.6. Vegetation Mapping

An important application in protected areas is the analysis of vegetation. In particular, monitoring the vegetal coverage is considered of great interest for the conservation authorities. In addition, taking advantage of the high resolution, classification at the species level is another objective to be pursued. In semi-arid ecosystems they are such challenging tasks, as the species are so small, are considerably affected by a significant soil reflectivity, are sparsely distributed, usually mixed and some of them have experienced phenological changes over time and space.

Vegetation has high absorption in the red and blue spectral regions and high reflectance in the near-infrared. Thus, many vegetation indices have been developed involving combinations of such solar-reflected spectral channels. Usually the red and near-infrared bands are considered in such a way that they strengthen the spectral contribution of green vegetation, minimizing the disturbing influence of soil background, irradiance, solar position, atmospheric attenuation, etc. [33,34].

In our analysis, the following 2 representative indices were assessed: Normalized Difference Vegetation Index (NDVI) and Enhanced Vegetation Index (EVI). NDVI involves the reflectance of near infrared (NIR) and red bands [35]:

(9) $$NDVI=\frac{\rho_{NIR}-\rho_{red}}{\rho_{NIR}+\rho_{red}}.$$

EVI directly adjusts the reflectance in the red band as a function of the reflectance in the blue band [36]:

(10) $$EVI=G\frac{\rho_{NIR}-\rho_{red}}{\rho_{NIR}+C_{1}\rho_{red}-C_{2}\rho_{blue}+L}\left(1+L\right),$$

where *L* is the canopy background adjustment and C1, C2 are the coefficients of the aerosol resistance term, which uses the blue band to correct for aerosol influences in the red band. The value of these coefficients are empirically determined as *L* = 1, C1 = 6, C2 = 7.5, and the gain *G* = 2.5.

Unfortunately, any index varies depending on the solar position, atmospheric attenuation, soil background season, hydric situation, etc. Thus, a fixed threshold cannot be set, even for the same sensor or season, to properly segment vegetation areas if a precise preprocessing of the satellite imagery has not been performed [37].

## 3. Results and Discussion

### 3.1. Variability of Model-Based Algorithms as Regards the Parametrization Setting

**Figure 6:**
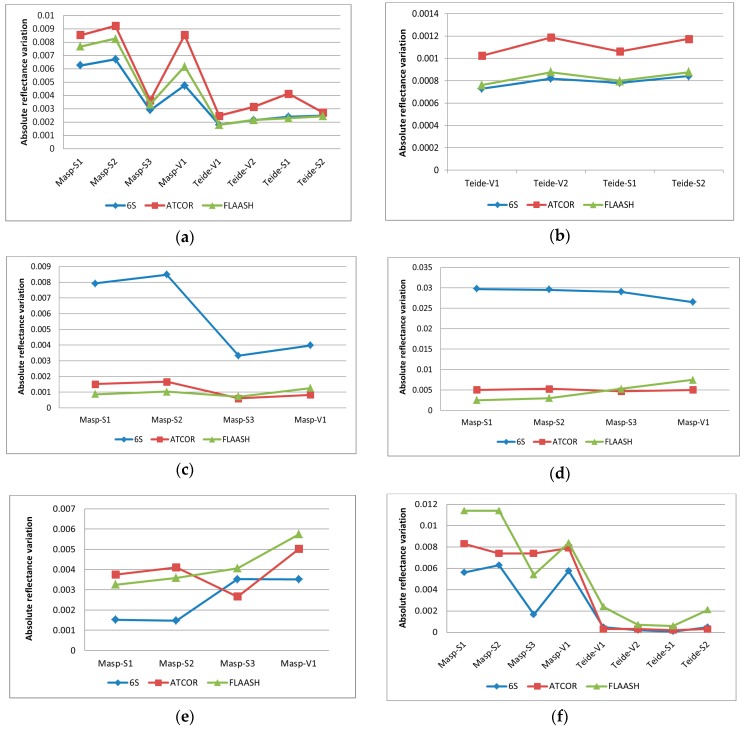
Absolute average reflectivity variation for Maspalomas (Masp) and Teide areas in soil (S) and vegetation (V) sites: (**a**) Tropical model with respect to the Mid-Latitude Summer model; (**b**) altitude of 2400 m with respect to 2250 m; (**c**) Rural aerosol model with respect to the Maritime model; (**d**) No aerosol model with respect to the Maritime model; (**e**) Maximum AOT (0.44) with respect the AOT of 0.25; (**f**) adjacency effect included with respect to the no inclusion of adjacency.

The effects of changing the parameter settings of the different physical models (FLAASH, ATCOR and 6S) are briefly detailed next for both areas of study. As regards the atmosphere model, the Mid-Latitude Summer seems the most suitable model for the climate of the Canary Islands. However, the Tropical model has also been included in the analysis as some summer months may be similar to this type of atmosphere. Water vapor was considered implicitly when selecting the atmosphere model as it considers standard column water vapor amounts (from sea level to space). For example, the Mid-Latitude Summer model accounts for an amount of 2.92 g/cm^2^ while the Tropical model is 4.11 g/cm^2^. Figure 6a shows that differences in the corrected reflectance are not too relevant for the three atmospheric algorithms (absolute average variation between 0.2% and 0.4% for Teide and from 0.4% to 0.9% for Maspalomas). Lower variations for the Teide region are consistent with the influence of the altitude in the water vapor column. ATCOR presents the highest variations while 6S the lowest.

In this sense, applying different altitudes at the scene has demonstrated minor variations in the estimated reflectance. In particular, for the Teide region, an average height of 2250 m with a range of variation from 2100 to 2400 was assumed. The absolute mean changes in both limits were less than 0.12% for ATCOR, and 0.08% for FLAASH and 6S (Figure 6b).

The most acceptable aerosol model for the islands is the Maritime model, but it has also been compared with the Rural model as well as the assumption of an atmosphere free of aerosols. 6S is more affected by the setting of this parameter. As regards to the Rural model (Figure 6c), the maximum variation by 6S, FLAASH and ATCOR methods are 0.85%, 0.13% and 0.16% respectively. When considering an atmosphere free of aerosols (Figure 6d), fluctuations are higher for 6S (values between 2.7% and 3.3%) while FLAASH and ATCOR are less affected (reflectivity variations of around 0.5%).

The aerosol optical thickness (AOT) parameter must be properly adjusted using in-situ or satellite information because major errors in their estimation can significantly affect the surface reflectivity computed. Nowadays, such information is daily available from satellite sensors (i.e., MODIS at 550 nm). In our case, AOT was recorded during the field campaign and also, for the Teide scene, precise data is available through the ground-based AERONET-CIAI-AEMET remote sensing aerosol network. Therefore, AOT was only slightly changed for the test, considering two values (044 and 0.25). In Maspalomas (Figure 6e), 6S is less affected with reflectivity variations among 0.15% to 0.35%, whereas ATCOR and FLAASH showed slightly higher variations in the mean absolute percentage values, but lower than 0.57%.

Finally, the inclusion of the adjacency effect (Figure 6f) does not generate considerable variations for the Teide samples (below 0.2%) but affects Maspalomas more (maximum change of around 1.1%). Specifically, FLAASH is more affected by this parameter while 6S has lower sensitivity. As presented, this result is scene-dependent as the adjacency effect is more important when the surrounding surface of a given pixel is different from the pixel itself.

In many applications, the appropriate setting is important for an accurate atmospheric correction. In our case, the analysis of the different input factors setting in the estimated reflectance of soil and vegetation sites led to slight changes as a consequence of the selection of the appropriate inputs for each area and date.

### 3.2. Absolute Evaluation Using in-Situ Spectroradiometer Measurements

The objective was to compare the estimated ground reflectance values of the corrected WV-2 Maspalomas image with the real in situ measurements over twelve ground points, as set out in Figure 5. The 5 atmospheric correction methods were considered in the analysis.

The most sensitive input factors used for FLAASH, ATCOR and 6S models have been derived from the relative analysis described in Section 3.1. After this analysis of the influence induced by each factor, and taking into account the typical available information, the four scenarios (A, B, C and D) presented in the Table 1 have been used to find the most suitable configuration. Adjacency effect, optical thickness and aerosol type are the parameters to be adjusted.

The overall results from the average measurements for all the vegetation and bare soil points analyzed are included in Table 2, in which the RMSE and BIAS between measured and corrected reflectance are presented. The results show that correction applying algorithms based on the physical modelling is more precise. These strategies obtain good estimations with RMSE values of between 2% and 3%, whereas image-based methods can only reach values of around 5%. The A scenario is more appropriate for the study area and, consequently, provides the best results.

**Figure 7:**
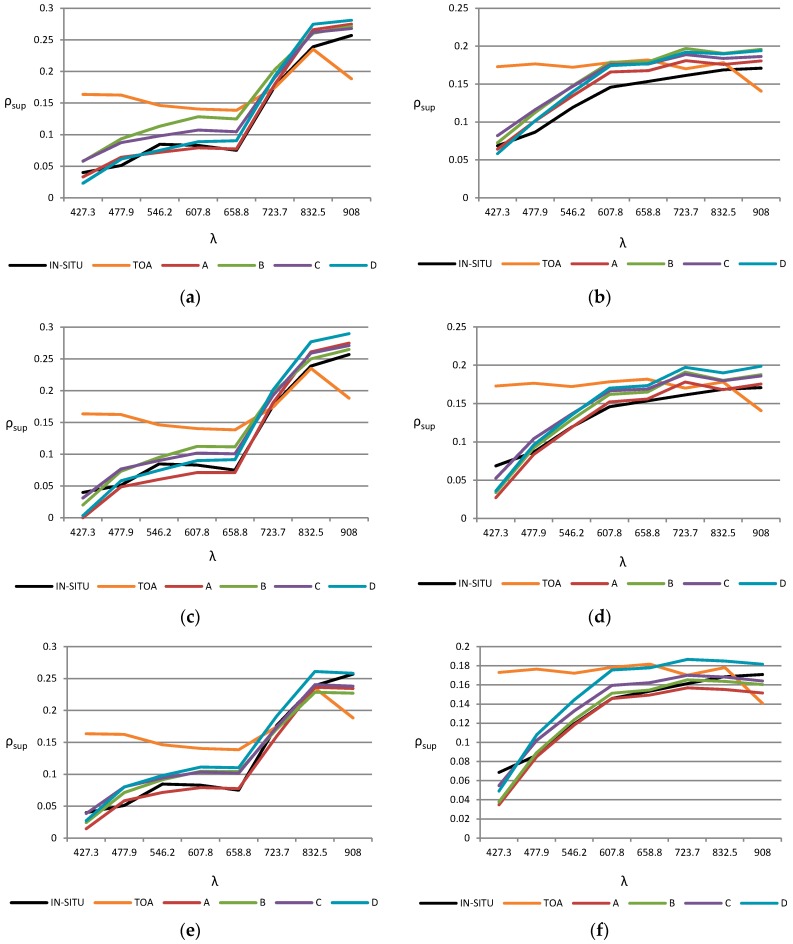
Spectral reflectivity signatures applying different atmospheric inputs for the following methods: (**a**) FLAASH at a vegetation point; (**b**) FLAASH at a bare soil point; (**c**) ATCOR at a vegetation point; (**d**) ATCOR at a bare soil point; (**e**) 6S at a vegetation point; (**f**) 6S at a bare soil point.

Results achieved by each model-based algorithm for the four scenarios, as well as the reference signature measured with the spectroradiometer (black) and the reflectance at the top of the atmosphere (orange) are plotted in Figure 7. The great impact of the reflectance corrections can be appreciated in the VIS bands and, in general, in shorter wavelengths bands. Only the signature of one representative vegetation and bare soil point are included on the left and right side, respectively, but similar conclusions can be derived from the remaining points analyzed.

In general, the spectral signature estimated by the atmospheric correction algorithms matches with the data from the in-situ measurements, except in the coastal blue channel where the reflectance is typically underestimated. Differences between the corrected reflectance for the four scenarios are relatively low, their being more significant when the adjacency effect is not included or the aerosol model is set to rural. Furthermore, the estimated reflectance of bare solid points is generally overestimated with the atmospheric correction based on FLAASH model in respect to the other methods.

**Figure 8:**
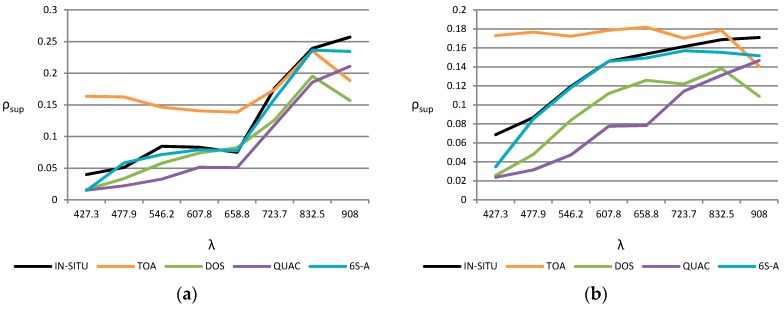
In situ, TOA and corrected spectral reflectivity signatures applying DOS, QUAC and 6S methods (A scenario): (**a**) vegetation point; (**b**) bare soil point.

As regards the results of the image-based algorithms, DOS and QUAC have a worse accuracy. Thus, Figure 8 shows the spectral signatures of representative bare soil and vegetation points. The results are compared with the in situ reflectance (black) and the TOA reflectivity (orange). The results obtained from the 6S model with the optimum configuration (A scenario) have also been included as a reference (blue). QUAC presents worse values for estimating the reflectivity.

In summary, after the analysis it is noted that for a good characterization of the reflectance values with FLAASH, ATCOR and 6S models, the adjacency effect, the suitable aerosol model of the scene and the appropriate AOT should be correctly included in their parametrization. The 6S algorithm provides the best overall accuracy with an improvement in the RMSE reflectivity of 0.6% and 1.1% compared to ATCOR and FLAASH respectively.

### 3.3. Vegetation Mapping

As previously mentioned, the atmospheric correction increases the discrimination potential of the classification process and it is of capital importance when dealing with multitemporal data since the viewing geometry, atmospheric conditions and plant phenology change from one date to another.

**Figure 9:**
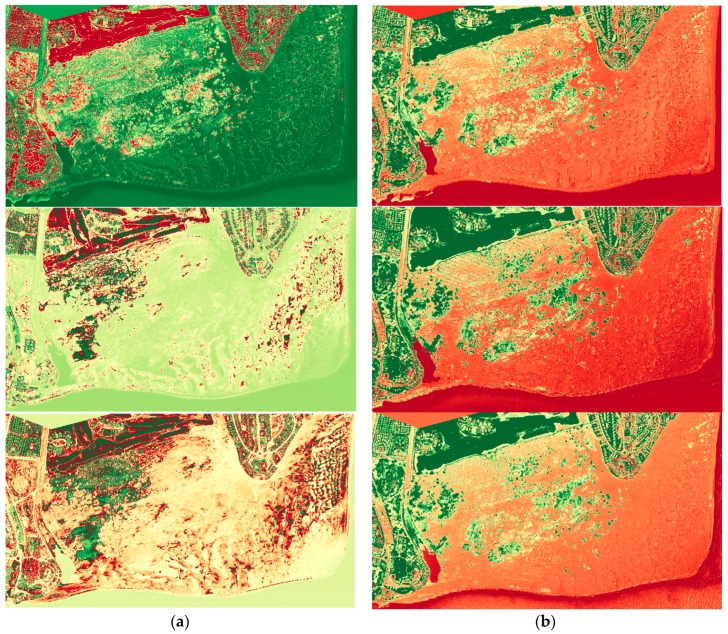
Enhanced Vegetation Index for three WV-2 subscenes of Maspalomas (17 January 2013, 11 August 2013 and 4 June 2015): (**a**) without atmospheric correction; (**b**) after the 6S correction.

As an example, Figure 9 shows the Enhanced Vegetation Index before and after applying the atmospheric correction for a multitemporal sequence of Maspalomas (to enhance the interpretation, a colorbar has been applied where greener tones indicate higher values of the index and dark reddish tones lower values). First, we can appreciate that vegetation is not properly discriminated in the three images when the atmospheric correction is not applied (left-hand column of Figure 9); while vegetation, soil and water covers are clearly identified after correction (right-hand column of Figure 9). Second, we can also check that after a precise correction, the three images sensed under different viewing and atmospheric conditions, look very similar having values for the EVI index in accordance with the land cover type.

As detailed in Equation (3), EVI uses the blue channel, thus vegetation detection requires an accurate atmospheric correction. Other vegetation indices, such as the NDVI index, will improve after the correction but not as significant as the EVI because the major impact of the reflectance correction can be noticed in bands near the blue and green regions.

**Figure 10:**
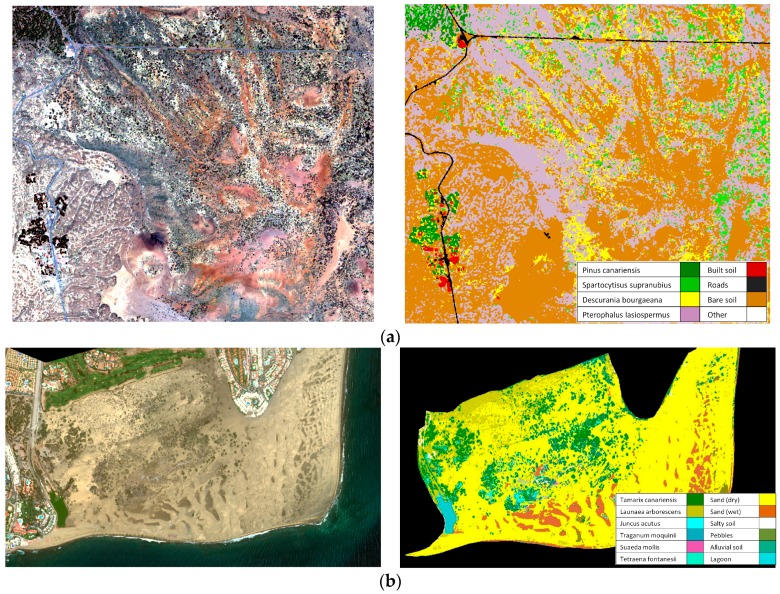
Vegetation maps using the SVM algorithm: (**a**) Teide National Park (16 May 2011); (**b**) Maspalomas Special Natural Reserve (4 June 2015).

Using WV-2 multispectral bands after the 6S atmospheric correction and the derived vegetation indices, the classification of vegetation cover was carried out by applying the supervised Support Vector Machines (SVM) algorithm with the radial basis function kernel [38,39]. SVM have proven to be very effective in solving complex classification problems, mainly due to the fact that this technique does not require an estimation of the statistical distribution of classes and the ability to handle limited amount or quality of training samples. Figure 10 shows thematic maps for both semi-arid regions. Using a database of well-known test regions, the overall accuracy achieved values of around 90% in both areas.

## 4. Conclusions

Atmospheric correction is a crucial step in the pre-processing of remote sensing imagery for many specific applications. The aim of this study was to assess the accuracy of atmospheric correction methodologies when applied to the mapping of vegetation with high resolution data. Specifically, this work carried out a comparison between five atmospheric correction algorithms (three model-based and two image-based) applied to the eight bands of the WV-2 satellite. The effects of corrections were studied in two types of protected semi-arid ecosystems (mountain and coastal). To evaluate the results, spectroradiometer in-situ data were collected simultaneously to the satellite overflight.

Two different analyses were conducted to compare the atmospheric correction methods. The first one addressed the study of the variations on the estimated reflectivity depending on the input configuration set for the algorithms based on the physical modelling of the atmosphere. Next, the second study consisted of a statistical assessment comparing the corrected spectral reflectivity of each technique with respect to in-situ measures. In this case, the high spatial resolution was exploited to compare the reflectance measured on the ground directly with the retrieved surface reflectance from the remote sensing data. This procedure allows the full correction algorithm to be validated including the combined effects of the aerosol optical thickness, water vapor, adjacency, etc.

In addition, examples of the improvement achieved by applying atmospheric correction techniques in the generation of multispectral and multitemporal vegetation indices have been presented. The use of these vegetation indices is important in protected areas to monitor the vegetation distribution and variability.

It was demonstrated that model-based algorithms achieve the best performance and, in particular, 6S reached the lowest value of the RMSE for both vegetation and bare soil areas. Consequently, it has been applied to the successful generation of challenging thematic maps in these protected areas.

The excellent results provided by these studies have been successfully applied to the generation of thematic maps in two challenging semi-arid ecosystems (the Teide and Maspalomas protected areas) and they are currently being used in the management of such natural reserves.

## Figures and Tables

**Table 1:** Scenarios considered for the comparison between satellite and field data.

Scenarios	A	B	C	D
Atmosphere model	Mid-Latitude Summer	Mid-Latitude Summer	Mid-Latitude Summer	Mid-Latitude Summer
Aerosol model	Maritime	Maritime	Maritime	Rural
AOT	0.44	0.44	0.25	0.44
Adjacency	Yes	No	Yes	Yes

AOT: Aerosol Optical Thickness.

**Table 2:** RMSE and BIAS between the in-situ measurements and satellite corrected reflectance for each atmospheric algorithm.

Category	Algorithm	Scenario	RMSE	BIAS
No correction	TOA reflectivity	-	0.0690	0.0430
Image-based	DOS	-	0.0465	0.0327
	QUAC	-	0.0578	0.0466
Physical model-based	FLAASH	A	**0.0333**	**0.0264**
		B	0.0442	0.0357
		C	0.0399	0.0346
		D	0.0398	0.0325
	ATCOR	A	**0.0290**	**0.0134**
		B	0.0362	0.0192
		C	0.0347	0.0259
		D	0.0406	0.0295
	6S	A	**0.0223**	**0.0021**
		B	0.0294	0.0056
		C	0.0289	0.0151
		D	0.0380	0.0281
